# Development of Powerly, unguided mobile app intervention preventing postpartum depression and anxiety & study protocol of randomized clinical trial

**DOI:** 10.1016/j.invent.2025.100843

**Published:** 2025-06-16

**Authors:** Marta A. Marciniak, Judith Rohde, Kenneth S.L. Yuen, Harald Binder, Henrik Walter, Matthias J. Wieser, Raffael Kalisch, Karin Roelofs, Birgit Kleim

**Affiliations:** a Department of Psychology, Education and Child Studies, Erasmus University Rotterdam, Rotterdam, the Netherlands; b Healthy Longevity Center, University of Zurich, Zurich, Switzerland; c Department of Psychology, University of Zurich, Zurich, Switzerland; d Department of Psychiatry, Psychotherapy and Psychosomatics, Psychiatric University Hospital, Zurich, Switzerland; e Neuroimaging Center (NIC), Focus Program Translational Neuroscience (FTN), Johannes Gutenberg University Medical Center, Mainz, Germany; f Leibniz Institute for Resilience Research (LIR), Mainz, Germany; g Institute of Medical Biometry and Statistics, Faculty of Medicine and Medical Center, University of Freiburg, Freiburg, Germany; h Freiburg Center for Data Analysis and Modelling, University of Freiburg, Freiburg, Germany; i Charité - Universitätsmedizin Berlin, Corporate Member of Freie Universität Berlin, Humboldt-Universität zu Berlin, Berlin Institute of Health, Berlin, Germany; j Donders Institute for Brain, Cognition, and Behaviour, Radboud University, Nijmegen, the Netherlands; k Behavioral Science Institute, Radboud University, Nijmegen, the Netherlands

**Keywords:** Pregnancy, Postpartum depression, Postpartum anxiety, Stress, Mobile health, Ecological momentary intervention

## Abstract

**Background:**

Up to 30 % of pregnant individuals experience high levels of stress. At the same time, 15–20 % of new mothers develop postpartum depression, and 25–35 % experience postpartum anxiety. Mobile applications have the potential to provide an accessible, scalable solution to these mental health challenges. However, previous evidence indicates that none of the commercially available apps for perinatal depression and anxiety have been rigorously evaluated through randomized clinical trials (RCTs), and the quality of these apps remains moderate. In response to this gap, we aim to develop and empirically evaluate Powerly, a mobile app intervention designed to prevent postpartum depression and anxiety.

**Methods:**

We will conduct a two-arm RCT with 140 healthy pregnant participants to assess the impact of Powerly use compared to care as usual (CAU). Powerly is based on cognitive behavioral therapy techniques and developed in consultation with stakeholders, including healthcare professionals and pregnant individuals. It will offer personalized psychological support tailored to users' needs for four weeks. Self-reported mental health assessments will be collected at baseline, after four weeks of app use, and six weeks postpartum.

**Discussion:**

We anticipate that participants using Powerly will demonstrate significant improvements in mental health outcomes, including reduced rates of postpartum depression, compared to the CAU group. Additionally, we expect positive changes in emotion regulation, resilience, and mother and child outcomes, such as enhanced maternal bonding and a more positive birth experience. If proved effective, Powerly can offer a scalable, publicly accessible solution for pregnant individuals in need.

**Trial registration:**

NCT06610552

## Highlights

• Powerly is an mHealth intervention aiming to prevent postpartum depression and anxiety.
• Powerly uses CBT-based techniques, offering personalized psychological support.
• The study will include 140 healthy pregnant participants in a two-arm RCT.
• 70 participants will receive access to Powerly, and 70 will receive care as usual.
• Outcome measures include mental health, emotion regulation, and mother-child bonding.

## 1 Introduction

### 1.1 Mental health in pregnancy and postpartum

Up to 30 % of individuals experience extremely high stress during pregnancy (Loomans et al., 2013), which leads to preterm birth in up to half of such cases, low birth weight of the children, and worse bond with a baby resulting in lower prenatal self-care and thus more health-related problems (Cheng et al., 2016; Lilliecreutz et al., 2016; Shapiro et al., 2013; Walsh et al., 2019). Additionally, nearly half of women experience traumatic childbirth (Alcorn et al., 2010), around 15–20 % of new mothers suffer from postpartum depression (Slomian et al., 2019; Stewart and Vigod, 2019; Wang et al., 2021), and 25 to 35 % of women experience postpartum anxiety (Radoš et al., 2018). This, however, indicates only a percentage of women undergoing screening for such mental health challenges, and, in reality, the numbers may be even higher.

Furthermore, there is a significant treatment gap and out of these diagnosed, only 22 % are connected to care services (Byatt et al., 2015), leaving many women without much-needed support. Many individuals cannot access professional therapy services, for instance, due to financial matters, feeling that their mental health problems are not severe enough to engage in help-seeking, or – in the postpartum – because they do not feel comfortable leaving the newborn alone (Feldman and Perret, 2023). It results in decreased health of the mother, lowered quality of her relationships, and disturbed baby's cognitive, emotional, and behavioral development (Dahlberg and Aune, 2013; Feldman and Perret, 2023; Slomian et al., 2019). Thus, addressing mental health challenges during pregnancy and postpartum – and their prevention – is of high importance. Mobile applications present a highly scalable support tool that can be accessed by individuals across diverse socioeconomic backgrounds and without geographical limitations.

### 1.2 Mobile-app interventions for pregnancy

The field of mobile apps is indeed rapidly expanding, with numerous applications available for pregnant and postpartum individuals. These apps are generally regarded as low-cost tools that can be used privately and accessed at any time, which is particularly valuable for new parents. Such interventions are also considered effective (Lewkowitz et al., 2024; Zhou et al., 2022) and a recent meta-analysis revealed that digital interventions for postpartum depression (including mobile apps, websites, telemedicine) achieved even greater effect sizes than in-person interventions (Ansaari et al., 2024).

However, most of the existing apps do not have elements of evidence-based psychological interventions. In their review, Evans et al. (2022) showed that out of 39 mobile apps designed for women with anxiety during pregnancy, 33 included only mind-body techniques (such as yoga or hypnotherapy), without any established psychological components. Furthermore, they noted that only one app on the market provided empirical evidence of its effectiveness, but it also offered no psychotherapeutic components, instead, it provided information and advice related to childcare. This finding was further corroborated by other meta-analysis and market search, which revealed that none of the commercially available apps for perinatal depression and anxiety had been empirically tested in a randomized controlled trial (RCT), further, these apps had overall moderate quality, implicating a room for improvement both in terms of efficacy and acceptability (Z. Tsai et al., 2022).

Therefore, we aim to develop and test Powerly - an empirically tested mobile app intervention for pregnant women, designed to prevent postpartum depression and anxiety. If proven effective, this app has the potential to be made publicly available to women in need.

### 1.3 Current intervention and research question

Powerly is a mental health mobile app, in which pregnant women can track their mood and level of stress and receive personalized psychological modules to increase their mental health and wellbeing. By integrating evidence-based content and cognitive behavioral techniques (CBT) with insights from pregnant and postpartum individuals and health professionals, Powerly offers a comprehensive psychological support. Full description of the development process and the app itself is presented in the Methods – “Intervention” section.

In this randomized clinical trial, we want to investigate Powerly's efficacy in terms of.

1) mental health symptoms, namely depressive symptoms, anxiety symptoms and level of stress,

2) emotion regulation and resilience outcomes, such as cognitive emotion regulation strategies, positive appraisal style, and self-efficacy,

3) mother & child outcomes, including mother-fetus/infant bond and satisfaction of birth, and Powerly's acceptability, indexed by the therapeutic alliance, app functionality rating as well as adherence.

In attempt to close the treatment gap, the app will be tested in conditions as close to real-life app use as possible. For instance, participants will not receive financial reimbursement for their time, and the control group will receive care as usual (CAU). If proven effective, this app will be made publicly available to women in need.

## 2 Methods

### 2.1 Intervention development

#### 2.1.1 Phase I – Literature review

As a first step, we conducted a comprehensive review of the current literature on the therapeutic components used in digital interventions for pregnancy and postpartum periods. This enabled us to identify and select the most effective components to be incorporated into the intervention, ensuring that it is grounded in evidence-based practices. Our findings regarding the most frequently used components were in line with recent review of Canfield et al. (2023) which includes both prevention-oriented and treatment studies. Those components were psychoeducation, (for instance, used for treatment of depressive symptoms in pregnancy: Canfield et al., 2023; Forsell et al., 2017; and during postpartum; Franco et al., 2024; as well as for depression and anxiety prevention in postpartum: Dol et al., 2022; Jiao et al., 2019 and prevention: Fisher et al., 2016) mindfulness (implemented, for instance, in prevention for depression high-risk group during pregnancy in Hassdenteufel et al., 2023; but also for mental health treatment in pregnancy: Qi et al., 2023; Yang et al., 2019), cognitive restructuring (used for treatment of mental health conditions in both pregnancy and postpartum in Danaher et al., 2023; treatment specifically in pregnancy: Forsell et al., 2017; and treatment specifically in postpartum: Pugh et al., 2016), self-efficacy (used for stress prevention during pregnancy: Y.-J. Tsai et al., 2018), and peer support (with examples to be found for treatment in both pregnancy and postpartum in Danaher et al., 2023; for postpartum depression prevention: Dennis et al., 2009; and for postpartum depression treatment: Gjerdingen et al., 2013; Kamalifard et al., 2013).

In this initial phase of app development, we chose to omit psychoeducation due to the abundance of existing and effective tools. We opted to include cognitive restructuring, particularly positive cognitive reappraisal (PCR). This decision was based on encouraging preliminary findings in the pregnancy-related studies and substantial evidence of its effectiveness in other varied populations and in both face-to-face and digital setting (for instance, Acee and Weinstein, 2010; Dryman and Heimberg, 2018; Gruber et al., 2013; Jamieson et al., 2016). At this stage, due to popularity of mindfulness and peer support, and due to mixed mental health outcomes, for instance in Hassdenteufel et al. (2023), we decided to discuss the possibility of incorporating these components into the app with stakeholders during later stages of app development.

Further, we conducted review focused on therapeutic components in broader contexts. By inferring potential effectiveness from studies conducted on other populations and through other modalities, such as face-to-face therapies, we aimed to integrate evidence-based strategies that might not yet be well-represented in digital interventions for this population. This comprehensive approach ensured that our intervention is both innovative and based on wide spectrum of therapeutic research. We identified several additional components that could potentially be included in Powerly, such as acceptance, meditation, relaxation, problem-solving, gratitude, distancing, and self-monitoring, for instance (Bakker and Rickard, 2018; Carissoli et al., 2015; Hur et al., 2018; Shrier and Spalding, 2017; Watts et al., 2013). To ensure their relevance and feasibility, we consulted with stakeholders regarding the inclusion of these components in Powerly. This additional search resulted also in identifying mental imagery, a cognitive component effective in reducing depressive symptoms and worry in general population (Blackwell et al., 2015; Holmes, Lang and Deeprose, 2009, Holmes, Blackwell, Burnett Heyes, Renner and Raes, 2016; Skodzik et al., 2017) but not widely researched in the pregnant populations. Yet, it has been proved effective in increasing mother-fetus bond (Kordi et al., 2016), decreasing levels of pregnancy stress (Flynn et al., 2016), or in changing health behaviors during pregnancy (Giacobbi Jr et al., 2021). This module is often offered with behavioral activation, a key CBT component effective in decreasing anhedonia, depressive symptoms and boosting activity among varied samples, including pregnant population, and with confirmed effectiveness when delivered via digital interventions (Bakker et al., 2018; Birney et al., 2016; Dahne et al., 2019; Dimidjian et al., 2006; Furukawa et al., 2018; Lejuez et al., 2001; Schlosser et al., 2017). Due to promising findings and our supporting data, we decided to include both these modules, mental imagery and behavioral activation, in Powerly.

#### 2.1.2 Phase II – Development of leading therapeutic agents

Following the literature review, we integrated PCR and mental imagery as primary therapeutic components within Powerly.

Positive cognitive reappraisal was selected as a key stress resilience factor (Carlson et al., 2012; Kalisch et al., 2015). It has been successfully implemented in various therapeutic settings and proven effective in diverse contexts, as indicated by previous literature (Morello et al., 2023; Riepenhausen et al., 2022). Furthermore, individuals who can flexibly apply PCR techniques in response to the varying emotional demands of stressful situations tend to experience lower levels of perceived stress (Marciniak et al., 2024a). Given that pregnancy and early motherhood are often perceived as particularly stressful periods (Loomans et al., 2013), incorporating this component is essential. Within Powerly, pregnant individuals will be prompted to recall a recent stressful event and generate three possible reappraisals for this event. These reappraisals might include identifying unexpected benefits, learning something from the event, or considering advice they would give to someone facing a similar challenge. This particular module has been tested in two RCTs on a population of young adults in Switzerland who initially had a lower tendency to use PCR. It was found to be effective in increasing the use of PCR and in reducing anxiety symptoms, and highly feasible as indicated by over 80 % adherence (Marciniak et al., 2023).

Mental imagery was implemented as the second leading therapeutic agents due to its high effectiveness in reducing depressive symptoms and other mental health challenges (Holmes, Lang and Deeprose, 2009, Holmes, Blackwell, Burnett Heyes, Renner and Raes, 2016). Within Powerly, we specifically included positive prospective mental imagery (Ji et al., 2017). In this module, women will be asked to envision a positive event that might occur in the near future and to create a detailed mental image of this event, engaging all their senses as if they were already experiencing it. This mental imagery training has been previously tested in an RCT with young Swiss adults, where it was found to be effective in reducing levels of perceived stress and depressive symptoms compared to an active control group (Marciniak et al., 2024b). Additionally, participants who created more vivid and accessible for themselves images, benefitted more from this training, regardless of the anticipation and pleasure of such images (Marciniak et al., 2022), rendering this module particularly of interest for early motherhood, which may involve many positive, yet ambiguous imaginations.

To expand the empirical evidence supporting the effectiveness of both leading therapeutic components, we conducted an additional study across four other countries (Germany, Israel, Netherlands, Poland) (Bögemann et al., 2023, Bögemann et al., 2024). This multi-national research effort aimed to validate the robustness of the interventions and to inform future studies using these components. It also underscores the potential impact of these interventions, reinforcing their inclusion in Powerly.

#### 2.1.3 Phase III – Participatory development and stakeholder consultations

In response to call for including end-users in development phase to increase the acceptability of interventions (Brotherdale et al., 2024), we decided to invite pregnant individuals to contribute to co-creation of Powerly. Please note that in this section, we use the terms “pregnant individuals” and “pregnant women” interchangeably, as all individuals who provided feedback identified themselves as women. Moving forward, we will aim to include a more diverse range of stakeholders.

Over 90 women from twelve countries (Austria, Bulgaria, Denmark, Germany, Greece, Italy, the Netherlands, Poland, Sweden, Switzerland, Ukraine, and the United Kingdom), aged 18–40 participated in this phase of app development. All participants were healthy and without a diagnosis of severe mental health disorder. They were recruited via an extended network of the authors and collaborators, as well as with online announcements and the snowballing method to ensure a broad international reach. We used various methods, including four types of surveys (qualitative surveys for name and design (*n* = 56) and general use of digital support tools during pregnancy (*n* = 43), quantitative survey for general health (*n* = 37), and mixed-method survey for mental health needs (n = 37)) with total of *n* = 87 unique responders, and individual semi-structured interviews (*n* = 10) to gather their insights about Powerly. Individuals involved in the interviews did not participate in the surveys but followed the same inclusion criteria and were recruited in the same manner. All interviews were conducted by the same researcher (MM) and focused on participants' mental health needs and adoption of digital tools with critical assessment of their content. Each participant was invited to review a selection of proposed therapeutic components identified in Phase I, which helped inform decisions regarding their inclusion. MM then conducted a thematic analysis of the interview data, using a data-driven approach based on the guidelines by Braun and Clarke (2006) and Ibrahim (2012). Additionally, during the interviews and within the surveys, women shared personal experiences during pregnancy and early postpartum periods, including moments of heightened stress, the support received from their immediate surroundings, and their interactions with medical professionals. For instance, the qualitative analysis of survey entries revealed that the most stressful periods during pregnancy include the first trimester, alongside anxiety-inducing events such as genetic testing and first ultrasound examinations. This was reported as the most stressful event by all of responders. Additionally, giving birth emerged as a significant source of stress for expectant mothers. The second trimester seems to be the most peaceful time during pregnancy, except for high-risk pregnancies. For postpartum women, the transition to motherhood posed significant challenges, with birth, breastfeeding, and the first weeks at home with a newborn emerging as the most stressful periods. The anticipation and uncertainty surrounding childbirth, coupled with the demands of breastfeeding, and adjusting to the responsibilities of caring for a newborn, contributed to heightened levels of stress and anxiety in all participants. According to survey participants, social support, particularly from partners and peers, played a pivotal role in mitigating stress and fostering emotional well-being during pregnancy. Expectant mothers who received ample support from their partners often reported lower levels of anxiety and greater overall satisfaction with their pregnancy experiences. Furthermore, participants were consulted regarding their usage of various apps before, during, and after pregnancy, shedding light on their preferences and use patterns in this regard. With ten interview participants, we discussed potential additional components to be included in Powerly, both in terms of therapeutic content as well as features improving engagement with the app. Nine women expressed interest in the mindfulness component, and six showed interest in gratitude. None of the participants suggested including peer support, noting that they already received sufficient support from their personal networks and social media. Two women expressed interest in psychoeducation, while eight others considered it redundant. Fifty-six women also contributed valuable feedback on several aspects, such as the app's name, logo, color palette, appearance of the main app dashboard, providing insights into its feasibility and user experience.

Furthermore, we conducted consultations with psychiatrists (*n* = 3) and psychotherapists (n = 3) who work regularly with patients experiencing postpartum mental health issues. We also engaged specialists providing mental health support specifically during in-vitro treatments (*n* = 2), fertility experts (n = 3), researchers specializing in maternal mental health (*n* = 5), and founders of startups offering services and products for pregnant individuals and those undergoing conception efforts (*n* = 4). These specialists were recruited through the extended professional networks of the authors and their collaborators, many of whom work in psychiatric hospitals. The connection to the start-up founders was established via professional services offered by the University of Zurich and facilitated further recruitment through a snowball sampling approach. Their shared experiences resulted in adding further components to Powerly.

#### 2.1.4 Phase IV – Development of additional components

After consulting with pregnant individuals and specialists in reproductive and mental health and conducting extensive literature reviews to ensure each proposed component's effectiveness in enhancing stress resilience or reducing mental health issues, we have chosen to incorporate four additional modules into Powerly.

Self-efficacy was identified as a key intervention component based on input from reproductive health specialists. Research suggests that higher self-efficacy is associated with lower perinatal anxiety, and greater stress resilience during pregnancy (Ma et al., 2021). In broader populations, reduced self-efficacy has also been linked to increased risk of negative mood and depressive symptoms (Rohde et al., 2023; Zhou et al., 2021). Evidence suggests that web-based self-efficacy training can effectively reduce pregnancy-related stress in antenatal women (Y.-J. Tsai et al., 2018). Similarly, a mobile app-based self-efficacy intervention for students was shown to decrease trait anxiety and hopelessness (Rohde et al., 2024). Within Powerly, users will be encouraged to recall a previous experience where they demonstrated mastery, overcame difficulty, or excelled in a challenging situation despite initial self-doubt.

Behavioral activation, recommended by mental health professionals and supported by existing literature, involves engaging in small, enjoyable activities to enhance mood. While it has been preliminarily shown to reduce depressive symptoms in pregnant and postpartum women experiencing elevated levels of depression (Dimidjian et al., 2017; O'Mahen et al., 2013), its effectiveness in a preventative context in this population remains largely unexplored (Mancinelli et al., 2023). Additionally, existing evidence suggests that current digital behavioral activation interventions for pregnant women often suffer from low adherence and high dropout rates (Mancinelli, Dell'Arciprete, Pattarozzi, Gabrielli and Salcuni, 2023, Mancinelli, Gabrielli and Salcuni, 2024), hence there is a need for a new tool implementing behavioral activation in this population. In a broader clinical context, behavioral activation encourages individuals to engage in rewarding activities, increasing positive reinforcement and disrupting the cycle of various mental health disorders, including, but not limited to, depression (Dimaggio and Shahar, 2017; Nagy et al., 2020). In Powerly, users will be encouraged to identify a simple, enjoyable five-minute activity that could improve their mood, fostering a habit of engaging in positive experiences.

Mindfulness was proposed by potential end-users as one of their favorite features in other apps. Digital mindfulness training has been shown to enhance mother-fetus bonding in healthy pregnant women (Patil and Malhotra, 2021). However, evidence regarding its effectiveness in alleviating perinatal stress, depression and anxiety, both in treatment and prevention contexts, remains mixed (Mefrouche et al., 2023; Taylor et al., 2016; Zhang et al., 2023), warranting a need for more research in this area. In this module, users will be guided through mindfulness exercise designed to enhance their awareness of both their inner experiences and their external environment. Women will be prompted to take a deep breath and focus on their current feelings and surroundings. They will be encouraged to observe the emotions evoked by their environment and to reflect on how much their feelings are influenced by the present moment. For example, they might be asked to notice the sensations of their breath, the sounds around them, or the textures they can feel, fostering a deeper connection to the here and now.

Gratitude was also put forward by our pregnant stakeholders. To the best of our knowledge, this module has rarely been tested in a preventative context for perinatal women and incorporated into digital mental well-being programs, with review by Corno et al. (2019) detecting only two such (online) interventions. However, research across various populations indicates that higher levels of gratitude are linked to improved mental well-being, life satisfaction, more positive emotions, and lower levels of stress, depression, and, in some studies, anxiety (Davis et al., 2016; Kirca et al., 2023; Komase et al., 2021; Sadeghi and Behzadi Pour, 2015). In this module, users will be instructed to reflect on three positive aspects of their lives. They will be encouraged to focus on either a specific event, such as favorable health examination results, or recurring positive elements, such as a loving partner, an understanding boss, good friends, or a healthy work-life balance.

**Fig. 1:**
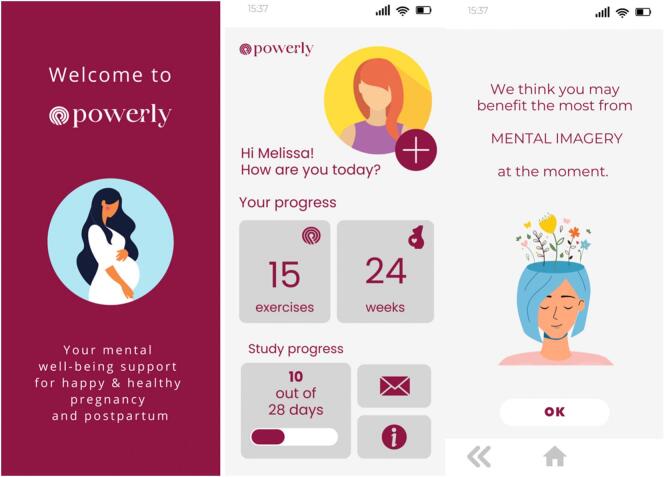
Example screens of Powerly app.

Example screens of Powerly are presented in Fig. 1. The final selection of the modules personalization features is presented in Table 1.

**Table 1:** Modules and personalization features included in the Powerly app.

Module	Personalization features
Reappraisal	Mood and affect tracking
Mental imagery	Name and icon selection
Self-efficacy	Progress tracking: number of completed exercises
Behavioral activation	Progress tracking: number of study days completed
Mindfulness	Pregnancy progress
Gratitude	Notifications

### 2.2 Study design

**Fig. 2:**
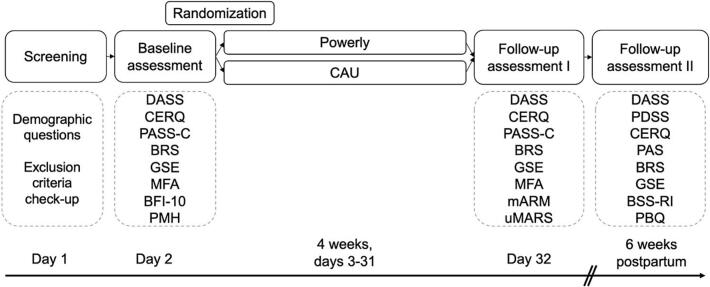
Study design and assessments.

We will conduct a two-arm RCT with repeated measures to evaluate the effects of Powerly compared to a control group receiving CAU. Assessments will occur at baseline, after four weeks of app use, and six weeks postpartum (Heller et al., 2020), see Fig. 2. The trial has been approved by the local ethics committee at the University of Zurich (approval #24.08.23) and has been registered with ClinicalTrials.gov (NCT06610552). The data collection will start in November 2024 until the desired sample size is reached. All procedures will be performed in accordance with the Declaration of Helsinki and its subsequent amendments. All participants will be asked to provide informed consent.

### 2.3 Participants

We will recruit participants mainly in Switzerland, Germany and Austria. Volunteers between 24 and 32 weeks pregnant will be included in the trial. Previous similar studies often included women in this range (Guo et al., 2020; Müller et al., 2020; Yang et al., 2019). Other inclusion criteria are being over the age of 18, owning an iPhone (due to technical limitations), and having sufficient fluency in German language. The exclusion criteria include high-risk pregnancies as self-reported, current substance abuse and history of psychiatric disorders such as bipolar disorder, schizophrenia, or other psychotic disorders, current use of professional face-to-face psychotherapeutic support, participation in other clinical trials or interventions at the same time.

### 2.4 Outcomes and measures

To ensure the selection of outcome measures was rigorous and scientifically relevant for the population of interest, we based our choices on recommendations from comprehensive reviews that specifically focus on state-of-the-art assessments of maternal mental health and the quality of the mother-child bond (Beck and Gable, 2001; Clarke et al., 2024; Perrelli et al., 2014; Sinesi et al., 2019; Sit and Wisner, 2009).

#### 2.4.1 Primary outcomes

The primary outcomes are related to mental health: (postpartum) depression, anxiety, and stress. These outcomes will be measured using the following validated instruments:

(a) DASS (Depression, Anxiety, and Stress Scales), a validated set of brief scales consisting of 21 items used to measure the severity of depression, anxiety, and stress symptoms (Lovibond and Lovibond, 1995),

(b) PDSS (Postpartum Depression Screening Scale), a comprehensive tool comprising 35 items designed to identify symptoms of postpartum depression, including aspects such as sleep and eating disturbances, insecurity, emotional lability, guilt, and shame (Beck and Gable, 2000).

#### 2.4.2 Secondary outcomes

Secondary outcomes are divided into three categories:

##### 2.4.2.1 Emotion regulation and resilience outcomes

(c) CERQ (Cognitive Emotion Regulation Questionnaire), which comprises 36 items assessing the strategies individuals employ to manage their emotions, including positive reappraisal, acceptance, rumination, and putting into perspective, among others (Garnefski and Kraaij, 2007).

(d) PASS-C (Positive Appraisal Style Scale – content-focused), comprises of fourteen items and measures person's general style of evaluating stressors (Petri-Romão et al., 2024).

(e) BRS (Brief Resilience Scale) measures resilience operationalized as an outcome of the process of adaptation to adversity, using five questions (Smith et al., 2008).

(f) GSE (General Self-Efficacy Scale) evaluates an individual's optimistic self-belief in their capability to handle difficult tasks, challenges, and adversities, through ten items (Schwarzer and Jerusalem, 1995).

##### 2.4.2.2 Mother & child outcomes

(g) BSS-RI (Birth Satisfaction Scale – Revised Indicator), which assesses the subjective experience of labor, including aspects of anxiety, stress, feelings of control, and support, using six items (Martin et al., 2017).

(h) MFA (Maternal-Fetal Attachment Scale), the 24-item scale evaluates the bond between mother and fetus during pregnancy. It includes questions about interactions with the fetus, attributing characteristics to the fetus, and role-taking, among others (Cranley, 1981). The MFA will be administered at baseline and the first follow-up.

(i) PBQ (Postpartum Bonding Scale), the 25-item questionnaire measures the quality of the bond between the mother and her infant. It includes factors such as the general bonding factor, anxiety about the baby, and rejection (Brockington et al., 2006). The PBQ will replace the MFA at the second follow-up.

##### 2.4.2.3 EMI Acceptability

(j) mARM (mobile Agnew Relationship Measure), which evaluates the therapeutic alliance between the app and the user through 25 items. It covers topics such as confidence, openness, user initiative, and partnership (Berry et al., 2018).

(k) uMARS (user version of the Mobile Application Rating Scale), tool used for assessment of the feasibility of the app, including aspects of engagement, aesthetics, functionality, and the quality of information provided in the EMI, with 20 questions (Stoyanov et al., 2016).

(l) Adherence – the number of interactions with the app by each of the participants, i.e., the number of completed entries in the app, and the total time spent on engagements with Powerly.

The baseline assessment will be complimented with (m) BFI-10 (Big Five Inventory-10), which assesses the five major dimensions of personality (openness, conscientiousness, extraversion, agreeableness, and neuroticism) with ten items (Rammstedt and John, 2007) and (n) PMH (Positive Mental Health scale) including nine items to measure specifically positive mental well-being (Lukat et al., 2016). The inclusion of these measures allows for exploratory analyses aimed at potentially customizing app delivery in the future research based on individual personality and mental well-being traits.

Refer to Fig. 2 for a detailed schematic representation of the study design and the timing of assessments.

### 2.5 Procedure

Participants will be recruited through multiple channels, primarily via gynecology wards in hospitals, gynecology and midwifery practices, and other pregnancy-related services such as birthing classes, prenatal yoga, and parenting workshops. To ensure a diverse sample with various backgrounds, we will also promote the study online through social media platforms targeting pregnant women. Volunteers interested in participating in the study will complete a brief online form providing basic demographic information (age, gender, education) and respond to questions regarding exclusion criteria. Those who meet the inclusion criteria will be contacted by a researcher and assigned a personal code for use throughout the study. The baseline assessment will be conducted online to accommodate participants who may have difficulties traveling while pregnant. Group allocation will be determined using a randomization algorithm generated by a National Institutes of Health clinical trial randomization tool, and the study personnel will be blinded to the group allocation. Participants in the intervention group after completing the baseline questionnaires, will receive instructions on how to download Powerly. They will be encouraged to use the app daily for 28 days, at least once per day, and will be invited to complete the questionnaires again. The duration and frequency of the intervention are based on previous research on CBT-based apps, which found that a four-week period with at least one interaction per day is both common and sufficient to achieve significant improvements in mental health outcomes while ensuring high adherence to the intervention and low drop-out rates (Birney et al., 2016; Marciniak et al., 2020). Participants in the CAU group will complete baseline questionnaires and be informed that they will receive access to Powerly six weeks postpartum. After 28 days, they will be invited to complete the questionnaires again, in a similar manner to Powerly group. Both groups will be contacted around their expected delivery date to confirm the birth date, and again six weeks postpartum for a final follow-up. After this, the CAU group will receive access to Powerly.

We do not anticipate any adverse effects caused by Powerly. However, to ensure early detection of any mental health distress in this vulnerable population, participants will have the option to contact the principal investigator via email throughout the trial. If participant's scores on the Postpartum Depression Screening Scale exceed the clinical threshold, they will be contacted and offered access to professional psychological services in their area.

### 2.6 Analysis

#### 2.6.1 Sample size calculation

The sample size calculation for linear mixed models was determined using Cohen's d = 0.5, a mean effect size observed in prior studies investigating digital interventions in pregnant women, where the estimates have ranged from d = 0.4 to d = 0.65 (Heller et al., 2020; Jiao et al., 2019; Qin et al., 2022; Sawyer et al., 2019; Shorey et al., 2019). With a power of 80 % and a significance level of 0.05, the anticipated sample size was 67 participants per group. To account for dropouts, based on our previous studies with similar mobile apps (Marciniak et al., 2024b; Marciniak et al., 2024c), we will aim to include 70 participants per group.

#### 2.6.2 Primary and secondary outcomes

The adherence will be determined as the sum of all interactions with the app by each study participant, specifically: the number of completed entries, and the total time spent on engagements with Powerly.

The questionnaire scores will be calculated according to the manuals, including the relevant subscales. The primary statistical method for other primary and secondary outcomes will be linear mixed models, with time points (baseline versus follow-up I versus follow-up II) and group (Powerly versus CAU) as fixed effects, and participants as random effects. We will employ intention-to-treat analyses, in which we will include all randomized participants, regardless of their app use, complemented by as-treated analyses, including participants who will complete at least one module per day. If necessary, data transformation of dependent variables will be performed based on visual inspection of the data to ensure that the model assumptions are met. Secondary outcome measures will be utilized both as independent variables to assess the effectiveness of Powerly on these outcomes, and as moderators to further explore the Powerly's effect on primary outcome measures. Other covariates which may be used in the analysis include age, number of children, financial situation and stressors encountered by the participants.

The analyses will be conducted using R in R Studio.

## 3 Discussion

This study aims to evaluate the effectiveness of the Powerly app in a population of pregnant women, focusing on three main areas: 1) mental health outcomes, specifically postpartum depression, anxiety, and stress; 2) emotion regulation and resilience outcomes, including positive appraisal style, cognitive emotion regulation strategies, and self-efficacy; and 3) mother & child related outcomes, such as maternal bonding and birth experience. Additionally, we will assess the acceptability of Powerly, measured by adherence, therapeutic alliance, and app quality ratings. We expect that participants using Powerly will show improvements in mental health outcomes, including lower postpartum depression rates, compared to CAU. We also anticipate positive changes in emotion regulation and resilience. In terms of mother & child outcomes, we expect improved maternal bonding and a more positive birth experience.

This study offers several clinical implications for maternal mental health care. If Powerly app proves effective in reducing mental health symptoms, it could offer a scalable, low-cost intervention that can be easily integrated into standard prenatal and postnatal care. Health care providers could recommend the app as part of a broader mental health strategy, offering support to women who may not have easy access to in-person therapy or resources. Additionally, the app's potential to improve emotion regulation, resilience, and maternal bonding could lead to better long-term outcomes for both mothers and their children. Finally, the study may provide valuable insights into how mobile health interventions can complement traditional care models, particularly in underserved populations.

However, there are certain limitations of this study, mostly related to the generalizability of the findings. For instance, high-risk pregnancies are not included in the trial. While these women may particularly benefit from such interventions, we have chosen to exclude them to first assess the potential for any adverse effects. Additionally, the absence of prior studies leaves potential adverse effects unknown, which adds some uncertainty to the app's potential unfavorable outcomes. Due to the technical constraints, we need exclude participants who do not own an iPhone, potentially missing a significant proportion of the population. In addition, participants who choose to enroll may already be more motivated to engage with digital interventions or have higher baseline mental health awareness, limiting generalizability to individuals who are less proactive about seeking support. While this study is the first trial to assess the feasibility and effectiveness of Powerly, future research could explore other study designs including, for instance, the use of a Sequential Multiple Assignment Randomized Trial (SMART) design (Almirall et al., 2014; Lei et al., 2012), to further optimize adaptive app delivery based on individual characteristics and response patterns.

Strengths of this study include testing in real-world conditions, allowing us to assess user engagement without additional incentives, and the use of CAU as a control condition, which will provide a clear comparison of the effects of Powerly against standard prenatal care. Another notable strength is the potential to reach underserved population and the app's capacity for future development to address other maternal mental health challenges, such as infertility, miscarriage, postpartum support, and couples' support during these challenges.

In conclusion, this randomized clinical trial is designed to generate rigorous evidence regarding the efficacy and acceptability of Powerly as a preventative intervention during pregnancy. The findings may help in advancing the quality of care available to pregnant individuals and inform future integration of mobile health tools into maternal healthcare frameworks.

## CRedit authorship contribution statement

MM, JR, and BK were involved in the development of Powerly. MM conceptualized the procedures. MM and HB defined the analysis. MM wrote the first version of the manuscript. All authors contributed to and approved the final version of the manuscript.

## Financial support

The basis for the project was developed during funding received from the European Union's Horizon 2020 research and innovation programme under grant agreement No. 777084 (DynaMORE project). MM was additionally supported by Digital Entrepreneur Fellowship (No. 22-003) funded by UZH Foundation, BRIDGE Proof of Concept (No. 222423) funded by Swiss National Science Foundation and Innosuisse, and SSH breed sectorplan, Netherlands.

This study reflects only the authors' view and the European Commission, UZH Foundation, Swiss National Science Foundation and Innosuisse are not responsible for any use that may be made of the information it contains.

## Declaration of competing interest

None.

## Data Availability

The self-report data will be anonymized and published in an openly accessible repository.

## References

[bb0005] Acee T.W., Weinstein C.E. (2010). Effects of a value-reappraisal intervention on statistics students’ motivation and performance. J. Exp. Educ..

[bb0010] Alcorn K.L., O’Donovan A., Patrick J.C., Creedy D., Devilly G.J. (2010). A prospective longitudinal study of the prevalence of post-traumatic stress disorder resulting from childbirth events. Psychol. Med..

[bb0015] Almirall D., Nahum-Shani I., Sherwood N.E., Murphy S.A. (2014). Introduction to SMART designs for the development of adaptive interventions: with application to weight loss research. Transl. Behav. Med..

[bb0020] Ansaari N., Rajan S.K., Kuruveettissery S. (2024). Efficacy of in-person versus digital mental health interventions for postpartum depression: meta-analysis of randomized controlled trials. J. Reprod. Infant Psychol..

[bb0025] Bakker D., Kazantzis N., Rickwood D., Rickard N. (2018). A randomized controlled trial of three smartphone apps for enhancing public mental health. Behav. Res. Ther..

[bb0030] Bakker D., Rickard N. (2018). Engagement in mobile phone app for self-monitoring of emotional wellbeing predicts changes in mental health: MoodPrism. J. Affect. Disord..

[bb0035] Beck C.T., Gable R.K. (2000). Postpartum depression screening scale: development and psychometric testing. Nurs. Res..

[bb0040] Beck C.T., Gable R.K. (2001). Comparative analysis of the performance of the postpartum depression screening scale with two other depression instruments. Nurs. Res..

[bb0045] Berry K., Salter A., Morris R., James S., Bucci S. (2018). Assessing therapeutic Alliance in the context of mHealth interventions for mental health problems: development of the Mobile Agnew relationship measure (mARM) questionnaire. J. Med. Internet Res..

[bb0050] Birney A.J., Gunn R., Russell J.K., Ary D.V. (2016). MoodHacker Mobile web app with email for adults to self-manage mild-to-moderate depression: randomized controlled trial. JMIR Mhealth Uhealth.

[bb0055] Blackwell S.E., Browning M., Mathews A., Pictet A., Welch J., Davies J., Watson P., Geddes J.R., Holmes E.A. (2015). Positive imagery-based cognitive Bias modification as a web-based treatment tool for depressed adults: a randomized controlled trial. Clin. Psychol. Sci..

[bb0060] Bögemann S., Krause F., van Kraaij A., Marciniak M., van Leeuwen J., Weermeijer J., Mituniewicz J., Puhlmann L., Zerban M., Reppmann Z., Kobylinska D., Yuen K., Kleim B., Walter H., Myin-Germeys I., Kalisch R., Roelofs K., Hermans E. (2024). Triggering just-in-time adaptive interventions based on real-time detection of daily-life stress. OSF.

[bb0065] Bögemann S.A., Riepenhausen A., Puhlmann L.M.C., Bar S., Hermsen E.J.C., Mituniewicz J., Reppmann Z.C., Uściƚko A., van Leeuwen J.M.C., Wackerhagen C., Yuen K.S.L., Zerban M., Weermeijer J., Marciniak M.A., Mor N., van Kraaij A., Köber G., Pooseh S., Koval P., Walter H. (2023). Investigating two mobile just-in-time adaptive interventions to foster psychological resilience: research protocol of the DynaM-INT study. BMC Psychology.

[bb0070] Braun V., Clarke V. (2006). Using thematic analysis in psychology. Qual. Res. Psychol..

[bb0075] Brockington I.F., Fraser C., Wilson D. (2006). The postpartum bonding questionnaire: a validation. Arch. Womens Ment. Health.

[bb0080] Brotherdale R., Berry K., Branitsky A., Bucci S. (2024). Co-producing digital mental health interventions: a systematic review. DIGITAL HEALTH.

[bb0085] Byatt N., Levin L.L., Ziedonis D., Moore Simas T.A., Allison J. (2015). Enhancing participation in depression Care in Outpatient Perinatal Care Settings: a systematic review. Obstet. Gynecol..

[bb0090] Canfield S.M., Canada K.E., Rolbiecki A.J., Petroski G.F. (2023). Feasibility and acceptability of an online mental health intervention for pregnant women and their partners: a mixed method study with a pilot randomized control trial. BMC Pregnancy Childbirth.

[bb0095] Carissoli C., Villani D., Riva G. (2015). Does a meditation protocol supported by a mobile application help people reduce stress? Suggestions from a controlled pragmatic trial. Cyberpsychol. Behav. Soc. Netw..

[bb0100] Carlson J.M., Dikecligil G.N., Greenberg T., Mujica-Parodi L.R. (2012). Trait reappraisal is associated with resilience to acute psychological stress. J. Res. Pers..

[bb0105] Cheng E.R., Park H., Wisk L.E., Mandell K.C., Wakeel F., Litzelman K., Chatterjee D., Witt W.P. (2016). Examining the link between women’s exposure to stressful life events prior to conception and infant and toddler health: the role of birth weight. J. Epidemiol. Community Health.

[bb0110] Clarke J.R., Gibson M., Savaglio M., Navani R., Mousa M., Boyle J.A. (2024). Digital screening for mental health in pregnancy and postpartum: a systematic review. Arch. Womens Ment. Health.

[bb0115] Corno G., Espinoza Macarena, Baños Maria R. (2019). A narrative review of positive psychology interventions for women during the perinatal period. J. Obstet. Gynaecol..

[bb0120] Cranley M.S. (1981). Development of a tool for the measurement of maternal attachment during pregnancy. Nurs. Res..

[bb0125] Dahlberg U., Aune I. (2013). The woman’s birth experience—the effect of interpersonal relationships and continuity of care. Midwifery.

[bb0130] Dahne J., Collado A., Lejuez C.W., Risco C.M., Diaz V.A., Coles L., Kustanowitz J., Zvolensky M.J., Carpenter M.J. (2019). Pilot randomized controlled trial of a Spanish-language behavioral activation mobile app (¡Aptívate!) for the treatment of depressive symptoms among United States Latinx adults with limited English proficiency. J. Affect. Disord..

[bb0135] Danaher B.G., Seeley J.R., Silver R.K., Tyler M.S., Kim J.J., La Porte L.M., Cleveland E., Smith D.R., Milgrom J., Gau J.M. (2023). Trial of a patient-directed eHealth program to ameliorate perinatal depression: the MomMoodBooster2 practical effectiveness study. Am. J. Obstet. Gynecol..

[bb0140] Davis D.E., Choe E., Meyers J., Wade N., Varjas K., Gifford A., Quinn A., Hook J.N., Van Tongeren D.R., Griffin B.J., Worthington E.L. (2016). Thankful for the little things: a meta-analysis of gratitude interventions. J. Couns. Psychol..

[bb0145] Dennis C.-L., Hodnett E., Kenton L., Weston J., Zupancic J., Stewart D.E., Kiss A. (2009). Effect of peer support on prevention of postnatal depression among high risk women: multisite randomised controlled trial. BMJ (Clinical Research Ed.).

[bb0150] Dimaggio G., Shahar G. (2017). Behavioral activation as a common mechanism of change across different orientations and disorders. Psychotherapy.

[bb0155] Dimidjian S., Goodman S.H., Sherwood N.E., Simon G.E., Ludman E., Gallop R., Welch S.S., Boggs J.M., Metcalf C.A., Hubley S., Powers J.D., Beck A. (2017). A pragmatic randomized clinical trial of behavioral activation for depressed pregnant women. J. Consult. Clin. Psychol..

[bb0160] Dimidjian S., Hollon S.D., Dobson K.S., Schmaling K.B., Kohlenberg R.J., Addis M.E., Gallop R., McGlinchey J.B., Markley D.K., Gollan J.K., Atkins D.C., Dunner D.L., Jacobson N.S. (2006). Randomized trial of behavioral activation, cognitive therapy, and antidepressant medication in the acute treatment of adults with major depression. J. Consult. Clin. Psychol..

[bb0165] Dol J., Aston M., Grant A., McMillan D., Tomblin Murphy G., Campbell-Yeo M. (2022). Effectiveness of the “essential coaching for every mother” postpartum text message program on maternal psychosocial outcomes: a randomized controlled trial. DIGITAL HEALTH.

[bb0170] Dryman M.T., Heimberg R.G. (2018). Emotion regulation in social anxiety and depression: a systematic review of expressive suppression and cognitive reappraisal. Clin. Psychol. Rev..

[bb0175] Evans K., Donelan J., Rennick-Egglestone S., Cox S., Kuipers Y. (2022). Review of Mobile apps for women with anxiety in pregnancy: maternity care professionals’ guide to locating and assessing anxiety apps. J. Med. Internet Res..

[bb0180] Feldman N., Perret S. (2023). Digital mental health for postpartum women: perils, pitfalls, and promise. NPJ Digit. Med..

[bb0185] Fisher J., Rowe H., Wynter K., Tran T., Lorgelly P., Amir L.H., Proimos J., Ranasinha S., Hiscock H., Bayer J., Cann W. (2016). Gender-informed, psychoeducational programme for couples to prevent postnatal common mental disorders among primiparous women: cluster randomised controlled trial. BMJ Open.

[bb0190] Flynn T.A., Jones B.A., Ausderau K.K. (2016). Guided imagery and stress in pregnant adolescents. Am. J. Occup. Ther..

[bb0195] Forsell E., Bendix M., Holländare F., Szymanska von Schultz B., Nasiell J., Blomdahl-Wetterholm M., Eriksson C., Kvarned S., Lindau van der Linden J., Söderberg E. (2017). Internet delivered cognitive behavior therapy for antenatal depression: a randomised controlled trial. J. Affect. Disord..

[bb0200] Franco P., Olhaberry M., Kelders S., Muzard A., Cuijpers P. (2024). Guided web app intervention for reducing symptoms of depression in postpartum women: results of a feasibility randomized controlled trial. Internet Interv..

[bb0205] Furukawa T., Imai H., Horikoshi M., Shimodera S., Hiroe T., Funayama T., Akechi T. (2018). Behavioral activation: is it the expectation or achievement, of mastery or pleasure that contributes to improvement in depression?. J. Affect. Disord..

[bb0210] Garnefski N., Kraaij V. (2007). The cognitive emotion regulation questionnaire: psychometric features and prospective relationships with depression and anxiety in adults. Eur. J. Psychol. Assess..

[bb0215] Giacobbi P., Symons Downs D., Haggerty T., Pidhorskyi S., Long D.L., Clemmer M., Steinman S.A., Olfert M.D., Kinnamon K., Rao N., Staggs H., Adjeroh D. (2021). Feasibility and acceptability of guided imagery to sequentially address multiple health behaviors during pregnancy. J. Midwifery Womens Health.

[bb0220] Gjerdingen D.K., McGovern P., Pratt R., Johnson L., Crow S. (2013). Postpartum doula and peer telephone support for postpartum depression: a pilot randomized controlled trial. J. Prim. Care Community Health.

[bb0225] Gruber J., Hay A.C., Gross J.J. (2013). Rethinking emotion: cognitive reappraisal is an effective positive and negative emotion regulation strategy in bipolar disorder. Emotion Advance online publication.

[bb0230] Guo L., Zhang J., Mu L., Ye Z. (2020). Preventing postpartum depression with mindful self-compassion intervention: a randomized control study. J. Nerv. Ment. Dis..

[bb0235] Hassdenteufel K., Müller M., Abele H., Brucker S.Y., Graf J., Zipfel S., Bauer A., Jakubowski P., Pauluschke-Fröhlich J., Wallwiener M., Wallwiener S. (2023). Using an electronic mindfulness-based intervention (eMBI) to improve maternal mental health during pregnancy: results from a randomized controlled trial. Psychiatry Res..

[bb0240] Heller H.M., Hoogendoorn A.W., Honig A., Broekman B.F., van Straten A. (2020). The effectiveness of a guided internet-based tool for the treatment of depression and anxiety in pregnancy (MamaKits online): randomized controlled trial. J. Med. Internet Res..

[bb0245] Holmes E.A., Blackwell S.E., Burnett Heyes S., Renner F., Raes F. (2016). Mental imagery in depression: phenomenology, potential mechanisms, and treatment implications. Annu. Rev. Clin. Psychol..

[bb0250] Holmes E.A., Lang T.J., Deeprose C. (2009). Mental imagery and emotion in treatment across disorders: using the example of depression. Cogn. Behav. Ther..

[bb0255] Hur J.-W., Kim B., Park D., Choi S.-W. (2018). A scenario-based cognitive behavioral therapy Mobile app to reduce dysfunctional beliefs in individuals with depression: a randomized controlled trial. Telemedicine and E-Health.

[bb0260] Ibrahim M. (2012). Thematic analysis: a critical review of its process and evaluation. West East J. Soc. Sci..

[bb0265] Jamieson J.P., Peters B.J., Greenwood E.J., Altose A.J. (2016). Reappraising stress arousal improves performance and reduces evaluation anxiety in classroom exam situations. Soc. Psychol. Personal. Sci..

[bb0270] Ji J.L., Holmes E.A., Blackwell S.E. (2017). Seeing light at the end of the tunnel: positive prospective mental imagery and optimism in depression. Psychiatry Res..

[bb0275] Jiao N., Zhu L., Chong Y.S., Chan W.-C.S., Luo N., Wang W., Hu R., Chan Y.H., He H.-G. (2019). Web-based versus home-based postnatal psychoeducational interventions for first-time mothers: a randomised controlled trial. Int. J. Nurs. Stud..

[bb0280] Kalisch R., Müller M.B., Tüscher O. (2015). A conceptual framework for the neurobiological study of resilience. Behav. Brain Sci..

[bb0285] Kamalifard M., Yavarikia P., Kheiroddin J.B., Pourmehr H.S., Iranagh R.I. (2013). The effect of peers support on postpartum depression: a single-blind randomized clinical trial. J. Caring Sci..

[bb0290] Kirca A., Malouff M., J., & Meynadier, J. (2023). The effect of expressed gratitude interventions on psychological wellbeing: a Meta-analysis of randomised controlled studies. Int. J. Appl. Posit. Psychol..

[bb0295] Komase Y., Watanabe K., Hori D., Nozawa K., Hidaka Y., Iida M., Imamura K., Kawakami N. (2021). Effects of gratitude intervention on mental health and well-being among workers: a systematic review. J. Occup. Health.

[bb0300] Kordi M., Fasanghari M., Asgharipour N., Esmaily H. (2016). Effect of guided imagery on maternal fetal attachment in nulliparous women with unplanned pregnancy. Journal of Midwifery and Reproductive Health.

[bb0305] Lei H., Nahum-Shani I., Lynch K., Oslin D., Murphy S.A. (2012). A “SMART” design for building individualized treatment sequences. Annu. Rev. Clin. Psychol..

[bb0310] Lejuez C.W., Hopko D.R., LePage J.P., Hopko S.D., McNeil D.W. (2001). A brief behavioral activation treatment for depression. Cogn. Behav. Pract..

[bb0315] Lewkowitz A.K., Whelan A.R., Ayala N.K., Hardi A., Stoll C., Battle C.L., Tuuli M.G., Ranney M.L., Miller E.S. (2024). The effect of digital health interventions on postpartum depression or anxiety: a systematic review and meta-analysis of randomized controlled trials. Am. J. Obstet. Gynecol..

[bb0320] Lilliecreutz C., Larén J., Sydsjö G., Josefsson A. (2016). Effect of maternal stress during pregnancy on the risk for preterm birth. BMC Pregnancy Childbirth.

[bb0325] Loomans E.M., van Dijk A.E., Vrijkotte T.G.M., van Eijsden M., Stronks K., Gemke R.J.B.J., Van den Bergh B.R.H. (2013). Psychosocial stress during pregnancy is related to adverse birth outcomes: results from a large multi-ethnic community-based birth cohort. Eur. J. Pub. Health.

[bb0330] Lovibond P.F., Lovibond S.H. (1995). The structure of negative emotional states: comparison of the depression anxiety stress scales (DASS) with the Beck depression and anxiety inventories. Behav. Res. Ther..

[bb0335] Lukat J., Margraf J., Lutz R., van der Veld W.M., Becker E.S. (2016). Psychometric properties of the positive mental health scale (PMH-scale). BMC Psychology.

[bb0340] Ma R., Yang F., Zhang L., Sznajder K.K., Zou C., Jia Y., Cui C., Zhang W., Zhang W., Zou N., Yang X. (2021). Resilience mediates the effect of self-efficacy on symptoms of prenatal anxiety among pregnant women: a nationwide smartphone cross-sectional study in China. BMC Pregnancy Childbirth.

[bb0345] Mancinelli E., Dell’Arciprete G., Pattarozzi D., Gabrielli S., Salcuni S. (2023). Digital behavioral activation interventions during the perinatal period: scoping review. JMIR Pediatrics and Parenting.

[bb0350] Mancinelli E., Gabrielli S., Salcuni S. (2024). A digital behavioral activation intervention (JuNEX) for pregnant women with subclinical depression symptoms: explorative co-design study. JMIR Hum. Factors.

[bb0355] Marciniak M.A., Homan S., Zerban M., Schrade G., Yuen K., Kobylinska D., Walter H., Wieser M.J., Hermans E., Shanahan L., Kalisch R., Kleim B. (2024). Positive cognitive reappraisal flexibility is associated with lower levels of perceived stress. OSF.

[bb0360] Marciniak M.A., Shanahan L., Binder H., Kalisch R., Kleim B. (2022). Positive prospective mental imagery characteristics in young adults and their associations with depressive symptoms. PsyArXiv.

[bb0365] Marciniak M.A., Shanahan L., Myin-Germeys I., Veer I.M., Yuen K.S.L., Binder H., Walter H., Hermans E.J., Kalisch R., Kleim B. (2024). Imager—a mobile health mental imagery-based ecological momentary intervention targeting reward sensitivity: a randomized controlled trial. Appl. Psychol. Health Well Being.

[bb0370] Marciniak M.A., Shanahan L., Rohde J., Schulz A., Wackerhagen C., Kobylińska D., Tuescher O., Binder H., Walter H., Kalisch R., Kleim B. (2020). Standalone smartphone cognitive behavioral therapy–based ecological momentary interventions to increase mental health: narrative review. JMIR Mhealth Uhealth.

[bb0375] Marciniak M.A., Shanahan L., Veer I., Walter H., Binder H., Hermans E., Timmer J., Tuescher O., Kalisch R., Kleim B. (2023). Reapp – an mHealth app increasing reappraisal: results from two randomized controlled trials. PsyArXiv.

[bb0380] Marciniak M.A., Shanahan L., Yuen K.S.L., Veer I.M., Walter H., Tuescher O., Kobylińska D., Kalisch R., Hermans E., Binder H., Kleim B. (2024). Burst versus continuous delivery design in digital mental health interventions: evidence from a randomized clinical trial. DIGITAL HEALTH.

[bb0385] Martin C.R., Hollins Martin C., Redshaw M. (2017). The birth satisfaction scale-revised Indicator (BSS-RI). BMC Pregnancy Childbirth.

[bb0390] Mefrouche M.L., Siegmann E.-M., Böhme S., Berking M., Kornhuber J. (2023). The effect of digital mindfulness interventions on depressive, anxiety, and stress symptoms in pregnant women: a systematic review and meta-analysis. Eur. J. Investig. Health Psychol. Educ..

[bb0395] Morello K., Schäfer S.K., Kunzler A.M., Priesterroth L.-S., Tüscher O., Kubiak T. (2023). Cognitive reappraisal in mHealth interventions to foster mental health in adults: a systematic review and meta-analysis. Frontiers in Digital Health.

[bb0400] Müller M., Matthies L.M., Goetz M., Abele H., Brucker S.Y., Bauer A., Graf J., Zipfel S., Hasemann L., Wallwiener M., Wallwiener S. (2020). Effectiveness and cost-effectiveness of an electronic mindfulness-based intervention (eMBI) on maternal mental health during pregnancy: the mindmom study protocol for a randomized controlled clinical trial. Trials.

[bb0405] Nagy G.A., Cernasov P., Pisoni A., Walsh E., Dichter G.S., Smoski M.J. (2020). Reward network modulation as a mechanism of change in behavioral activation. Behav. Modif..

[bb0410] O’Mahen H.A., Woodford J., McGinley J., Warren F.C., Richards D.A., Lynch T.R., Taylor R.S. (2013). Internet-based behavioral activation--treatment for postnatal depression (Netmums): a randomized controlled trial. J. Affect. Disord..

[bb0415] Patil M., Malhotra J. (2021). Mindful digital program–based interventions and their role in pregnancy and fetal outcomes. Journal of South Asian Federation of Obstetrics and Gynaecology.

[bb0420] Perrelli J.G.A., Zambaldi C.F., Cantilino A., Sougey E.B. (2014). Mother-child bonding assessment tools. Rev. Paul. Pediatr..

[bb0425] Petri-Romão P., Engen H., Rupanova A., Puhlmann L., Zerban M., Neumann R.J., Malyshau A., Ahrens K.F., Schick A., Kollmann B., Wessa M., Walker H., Plichta M.M., Reif A., Chmitorz A., Tuescher O., Basten U., Kalisch R. (2024). Self-report assessment of positive appraisal style (PAS): development of a process-focused and a content-focused questionnaire for use in mental health and resilience research. PLoS One.

[bb0430] Pugh N.E., Hadjistavropoulos H.D., Dirkse D. (2016). A randomised controlled trial of therapist-assisted, internet-delivered cognitive behavior therapy for women with maternal depression. PLoS One.

[bb0435] Qi W., Zhao F., Huang S., Wei Z., Yang H., He K., Li C., Guo Q., Hu J. (2023). Effects and feasibility of a mindfulness-based Guqin music intervention during pregnancy on postpartum anxiety and depression: a pilot randomized controlled trial. Mindfulness.

[bb0440] Qin X., Liu C., Zhu W., Chen Y., Wang Y. (2022). Preventing postpartum depression in the early postpartum period using an app-based cognitive behavioral therapy program: a pilot randomized controlled study. Int. J. Environ. Res. Public Health.

[bb0445] Radoš S.N., Tadinac M., Herman R. (2018). Anxiety during pregnancy and postpartum: course, predictors and comorbidity with postpartum depression. Acta Clin. Croat..

[bb0450] Rammstedt B., John O.P. (2007). Measuring personality in one minute or less: a 10-item short version of the big five inventory in English and German. J. Res. Pers..

[bb0455] Riepenhausen A., Wackerhagen C., Reppmann Z.C., Deter H.-C., Kalisch R., Veer I.M., Walter H. (2022). Positive cognitive reappraisal in stress resilience, mental health, and well-being: a comprehensive systematic review. Emot. Rev..

[bb0460] Rohde J., Marciniak M.A., Henninger M., Homan S., Paersch C., Egger S.T., Seifritz E., Brown A.D., Kleim B. (2023). Investigating relationships among self-efficacy, mood, and anxiety using digital technologies: randomized controlled trial. JMIR Formative Research.

[bb0465] Rohde J., Marciniak M.A., Henninger M., Homan S., Ries A., Paersch C., Friedman O., Brown A.D., Kleim B. (2024). Effects of a digital self-efficacy training in stressed university students: a randomized controlled trial. PLoS One.

[bb0470] Sadeghi A., Behzadi Pour S. (2015). The effect of gratitude on psychological and subjective well-being among hospital staff. Health Education and Health Promotion.

[bb0475] Sawyer A., Kaim A., Le H.-N., McDonald D., Mittinty M., Lynch J., Sawyer M. (2019). The effectiveness of an app-based nurse-moderated program for new mothers with depression and parenting problems (eMums plus): pragmatic randomized controlled trial. J. Med. Internet Res..

[bb0480] Schlosser D.A., Campellone T.R., Truong B., Anguera J.A., Vergani S., Vinogradov S., Arean P. (2017). The feasibility, acceptability, and outcomes of PRIME-D: a novel Mobile intervention treatment for depression. Depress. Anxiety.

[bb0485] Schwarzer R., Jerusalem M. (1995). General Self-Efficacy Scale..

[bb0490] Shapiro G.D., Fraser W.D., Frasch M.G., Séguin J.R. (2013). Psychosocial stress in pregnancy and preterm birth: associations and mechanisms. J. Perinat. Med..

[bb0495] Shorey S., Chee C.Y.I., Ng E.D., Lau Y., Dennis C.-L., Chan Y.H. (2019). Evaluation of a technology-based peer-support intervention program for preventing postnatal depression (part 1): randomized controlled trial. J. Med. Internet Res..

[bb0500] Shrier L.A., Spalding A. (2017). “Just take a moment and breathe and think”: young women with depression talk about the development of an ecological momentary intervention to reduce their sexual risk. J. Pediatr. Adolesc. Gynecol..

[bb0505] Sinesi A., Maxwell M., O’Carroll R., Cheyne H. (2019). Anxiety scales used in pregnancy: systematic review. BJPsych Open.

[bb0510] Sit D.K., Wisner K.L. (2009). The identification of postpartum depression. Clin. Obstet. Gynecol..

[bb0515] Skodzik T., Leopold A., Ehring T. (2017). Effects of a training in mental imagery on worry: a proof-of-principle study. J. Anxiety Disord..

[bb0520] Slomian J., Honvo G., Emonts P., Reginster J.-Y., Bruyère O. (2019). Consequences of maternal postpartum depression: a systematic review of maternal and infant outcomes. Women Health.

[bb0525] Smith B.W., Dalen J., Wiggins K., Tooley E., Christopher P., Bernard J. (2008). The brief resilience scale: assessing the ability to bounce back. Int. J. Behav. Med..

[bb0530] Stewart D.E., Vigod S.N. (2019). Postpartum depression: pathophysiology, treatment, and emerging therapeutics. Annu. Rev. Med..

[bb0535] Stoyanov S.R., Hides L., Kavanagh D.J., Wilson H. (2016). Development and validation of the user version of the Mobile application rating scale (uMARS). JMIR Mhealth Uhealth.

[bb0540] Taylor B.L., Cavanagh K., Strauss C. (2016). The effectiveness of mindfulness-based interventions in the perinatal period: a systematic review and Meta-analysis. PLoS One.

[bb0545] Tsai Y.-J., Hsu Y.-Y., Hou T.-W., Chang C.-H. (2018). Effects of a web-based antenatal care system on maternal stress and self-efficacy during pregnancy: a study in Taiwan. J. Midwifery Womens Health.

[bb0550] Tsai Z., Kiss A., Nadeem S., Sidhom K., Owais S., Faltyn M., Lieshout R.J.V. (2022). Evaluating the effectiveness and quality of mobile applications for perinatal depression and anxiety: a systematic review and meta-analysis. J. Affect. Disord..

[bb0555] Walsh K., McCormack C.A., Webster R., Pinto A., Lee S., Feng T., Krakovsky H.S., O’Grady S.M., Tycko B., Champagne F.A., Werner E.A., Liu G., Monk C. (2019). Maternal prenatal stress phenotypes associate with fetal neurodevelopment and birth outcomes. Proc. Natl. Acad. Sci..

[bb0560] Wang Z., Liu J., Shuai H., Cai Z., Fu X., Liu Y., Xiao X., Zhang W., Krabbendam E., Liu S., Liu Z., Li Z., Yang B.X. (2021). Mapping global prevalence of depression among postpartum women. Transl. Psychiatry.

[bb0565] Watts S., Mackenzie A., Thomas C., Griskaitis A., Mewton L., Williams A., Andrews G. (2013). CBT for depression: a pilot RCT comparing mobile phone vs. computer. BMC Psychiatry.

[bb0570] Yang M., Jia G., Sun S., Ye C., Zhang R., Yu X. (2019). Effects of an online mindfulness intervention focusing on attention monitoring and acceptance in pregnant women: a randomized controlled trial. J. Midwifery Womens Health.

[bb0575] Zhang X., Li Y., Wang J., Mao F., Wu L., Huang Y., Sun J., Cao F. (2023). Effectiveness of digital guided self-help mindfulness training during pregnancy on maternal psychological distress and infant neuropsychological development: randomized controlled trial. J. Med. Internet Res..

[bb0580] Zhou C., Hu H., Wang C., Zhu Z., Feng G., Xue J., Yang Z. (2022). The effectiveness of mHealth interventions on postpartum depression: a systematic review and meta-analysis. J. Telemed. Telecare.

[bb0585] Zhou C., Yue X.D., Zhang X., Shangguan F., Zhang X.Y. (2021). Self-efficacy and mental health problems during COVID-19 pandemic: a multiple mediation model based on the health belief model. Personal. Individ. Differ..

